# Discordance of peripheral artery disease diagnosis using exercise transcutaneous oxygen pressure measurement and post-exercise ankle-brachial index

**DOI:** 10.1038/s41598-020-64276-x

**Published:** 2020-05-04

**Authors:** G. Mahé, F. Catillon, Q. Tollenaere, P. Jéhannin, A. Guilcher, E. Le Pabic, G. Lesager, L. Omarjee, A. Le Faucheur

**Affiliations:** 1 Vascular Medicine Unit, CHU, Rennes, France; 2 Univ Rennes 1; INSERM CIC 1414, Rennes, France; 3 CHU Rennes, Inserm, CIC 1414 (Clinical Investigation Center), F-35000, Rennes, France; 4 Vascular Medicine, Hospital, Redon, France; 5 Ecole Normale Supérieure, Bruz, France; 6 Univ Rennes, M2S – EA 7470, F-35000, Rennes, France

**Keywords:** Cardiovascular diseases, Peripheral vascular disease

## Abstract

In patients with exertional limb symptoms and normal ankle-brachial index (ABI) at rest, exercise testing can be used to diagnose lower extremity arterial disease (LEAD). Post-exercise ABI decrease or Exercise transcutaneous oxygen pressure measurement (Exercise-TcPO2) can be used to diagnose LEAD. Objectives were (i) to assess the agreement between both methods (ii) to define the variables associated with the discordance, and (iii) to present results of healthy subjects. In this prospective cross-sectional study, patients with exertional limb symptoms and normal rest ABI were consecutively included. ABI was measured at rest and after standardized exercise protocol as well as Exercise-TcPO2. A kappa coefficient with a 95% confidence interval was used to assess the agreement between the two methods. Logistic regression analysis was performed to outline variables potentially responsible for discordance. Ninety-six patients were included. The agreement between the tests was weak with a k value of 0.23 [0.04–0.41]. Logistic regression analysis found that a medical history of lower extremity arterial stenting (odds ratio 5.85[1.68–20.44]) and age (odds ratio 1.06[1.01–1.11]) were the main cause of discordance. This study suggests that post-exercise ABI and Exercise-TcPO2 cannot be used interchangeably for the diagnosis of LEAD in patients with exertional symptoms and normal rest ABI.

## Introduction

Lower extremity arterial disease (LEAD) is a highly prevalent disease with more than 200 million patients affected worldwide^1^ with only half presenting exertional limb symptoms^2^. The measurement of the ankle-brachial pressure index at rest (ABI) is the gold standard method for diagnosis of LEAD, with a value greater than 0.90 being considered normal^3,4^. However, in some cases, ABI can be normal in spite of the presence of exertional limb symptoms. In these cases, international guidelines recommend to perform other tests, including exercise tests^3–5^.

Post-Exercise ABI decrease (Post-Exercise ABI > 20% compared with baseline values or Post-Exercise pressure decrease >30 mmHg) has been proposed by the American Heart Association (AHA) as a second line tool to diagnose LEAD^3–5^. However, several studies have questioned the validity of the cut-off values currently used^6–9^.

Exercise transcutaneous oxygen pressure (Exercise TcPO2) is an alternative to Post-Exercise ABI to diagnose LEAD^8,10–12^. In the eighties, several groups have performed TcPO2 after exercise and during exercise without standardized methodology^13–16^. In 2003, Abraham and colleagues have proposed to perform Exercise TcPO2 with a standardized methodology (i.e. the places of the probe were defined, the room temperature was controlled and the duration of the pre-test heating period was defined)^10^. This standardized methodology associated with the use of the delta from rest of oxygen pressure (DROP) cut-off has improved the reliability of this procedure that was confirmed by two studies^17,18^. Finally, two different teams found similar cut-off values of the DROP^8,10,12,19^.

Stivalet *et al*. have recently shown that Post-Exercise ABI and Exercise-TcPO2 have similar accuracies to diagnose LEAD in patients with a normal resting ABI using computed tomography angiography (CTA) as a reference^8^. Indeed, the accuracy for the Post-exercise ABI was 67%[53–78] with a sensitivity of 71%[48–89] and specificity of 64%[47–79] whereas the accuracy of Exercise TcPO2 was 72%[59–83] with a sensitivity of 48%[26–70%]and a specificity of 85%[70–94]^8^.

A recent study has suggested that discordance of diagnosis of LEAD (20.8%) exits between exercise tests (Exercise TcPO2 and post-exercise pressure measurement) and ABI at rest in patients with normal and abnormal ABI^20^. However, there are several issues about the study: i) the authors did not detail how they did the pressure measurements at rest and after exercise; ii) they did not clarify if the ABI at rest was performed on the same day of the exercise tests; iii) they did not use the same exercise duration for the two exercise tests and, iv) they mixed the results of post-exercise ABI and post-exercise ankle pressure in the analyses. For all these reasons, additional study is required to assess the concordance between Exercise-TcPO2 and post-exercise pressure measurements in patients with exertional limb symptoms and normal ankle-brachial index (ABI).

Furthermore, although several studies have assessed the interest of exercise tests in suspected LEAD patients^10,12,21,22^, no study has reported healthy subject results of exercise tests (Exercise TcPO2 and post-exercise ABI after a treadmill test (10% slope and 3.2 km/h)).

Therefore, the aims of this study were i) to assess the concordance of Post-Exercise ABI and Exercise-TcPO2 drops to diagnose LEAD in patients with exertional limb symptoms and normal resting ABI, ii) to define the variables associated independently with the discordance, and iii) to show exercise test results obtained in healthy subjects.

## Results

**Figure 1:**
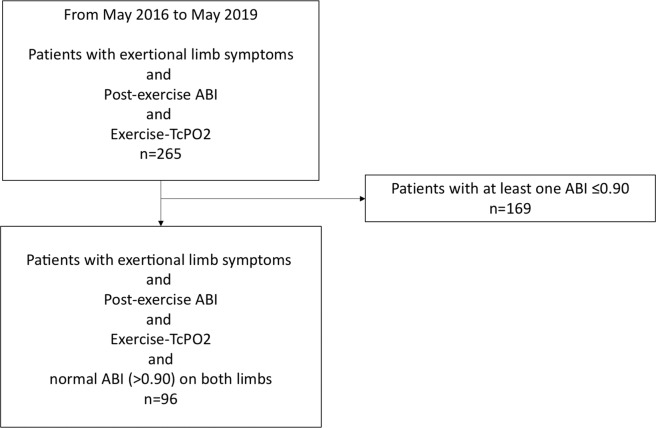
Diagram flow of the included population. Post-Exercise ABI means post-exercise ankle brachial index; Exercise-TcPO2 means exercise transcutaneous oxygen pressure measurement.

Among 265 consecutive patients who visited the clinic for standard care, 96 patients with exertional limb symptoms and normal ABI (>0.90) at rest on both limbs and who had performed both exercise tests were included (Fig. 1). Patient characteristics are presented in Table 1. The average age and body mass index were 61 +/−13 years, 26.2 +/−4.5 kg/m^2^, respectively. In this population, median estimated walking distance was 500 m [250–2500]. Median maximal walking distance on treadmill was 258 m [139–525] and 213 m [122–524] for Exercise-TcPO2 and for Post-Exercise ABI, respectively (p < 0.05). Mean heart rate before the first test (Exercise-TcPO2) was 76[68–86] beats per minute (bpm) and 81[71–93] bpm before the second test (Post-Exercise ABI) (p < 0.05).

**Table 1:** Characteristics of the population studied.

Clinical characteristics	*n* = 96
Age, years	61 ± 13
Male sex, no. (%)	73 (76%)
Body mass index, kg/m^2^	26.2 ± 4.5
Comorbidities, (history of), no. (%)	
Smoker (current or former)	76 (80%)
Coronary disease	25 (26%)
Hypercholesterolemia	58 (60%)
Diabetes	19 (20%)
Peripheral artery disease*	23 (24%)
Hypertension	53 (55%)
Stroke	6 (6%)
Current medications, no. (%)	
Statins	49 (51%)
Anti-coagulants	11 (12%)
Antiplatelet	58 (60%)
Others anticholesterolaemia	1 (1%)
Angiotensin-converting enzyme inhibitors	24 (25%)
Angiotensin II receptor antagonists	14 (15%)
Ankle brachial index at rest (right)	1.05 [0.97–1.14]
Ankle brachial index at rest (left)	1.03 [0.96–1.16]
Maximal walking distance self-reported, m	500 [250–2500]
Maximal treadmill walking distance (Exercise TcPO2), m	258 [139–525]
Maximal treadmill walking distance (Post-Exercise ABI), m	213 [122–524]

Results are presented mean ± standard deviation or median [25th ;75th], or number of observation (%). TcP02 = Transcutaneous oxygen pressure measurement. Post-Exercise ABI = Post-exercise ankle-brachial index. Peripheral artery disease* means history of revascularization (bypass/angioplasty/stent).

In the suspected LEAD population, the mean post-exercise ABI for the symptomatic leg was 0.87 +/−0.32. The mean post-exercise ABI decrease for the symptomatic leg was 21.6%+/−20.2%. The maximum post-exercise increase for the symptomatic leg was 14.3% and the maximal post-exercise decrease was 66.3%. Regarding Exercise TcPO2 values, the mean right DROP was −13 +/−12 mmHg whereas the mean left DROP was −13 +/−13 mmHg. The lowest DROP value was −79 mmHg and the maximal DROP value was 11 mmHg.

### Concordance between both tests

**Figure 2:**
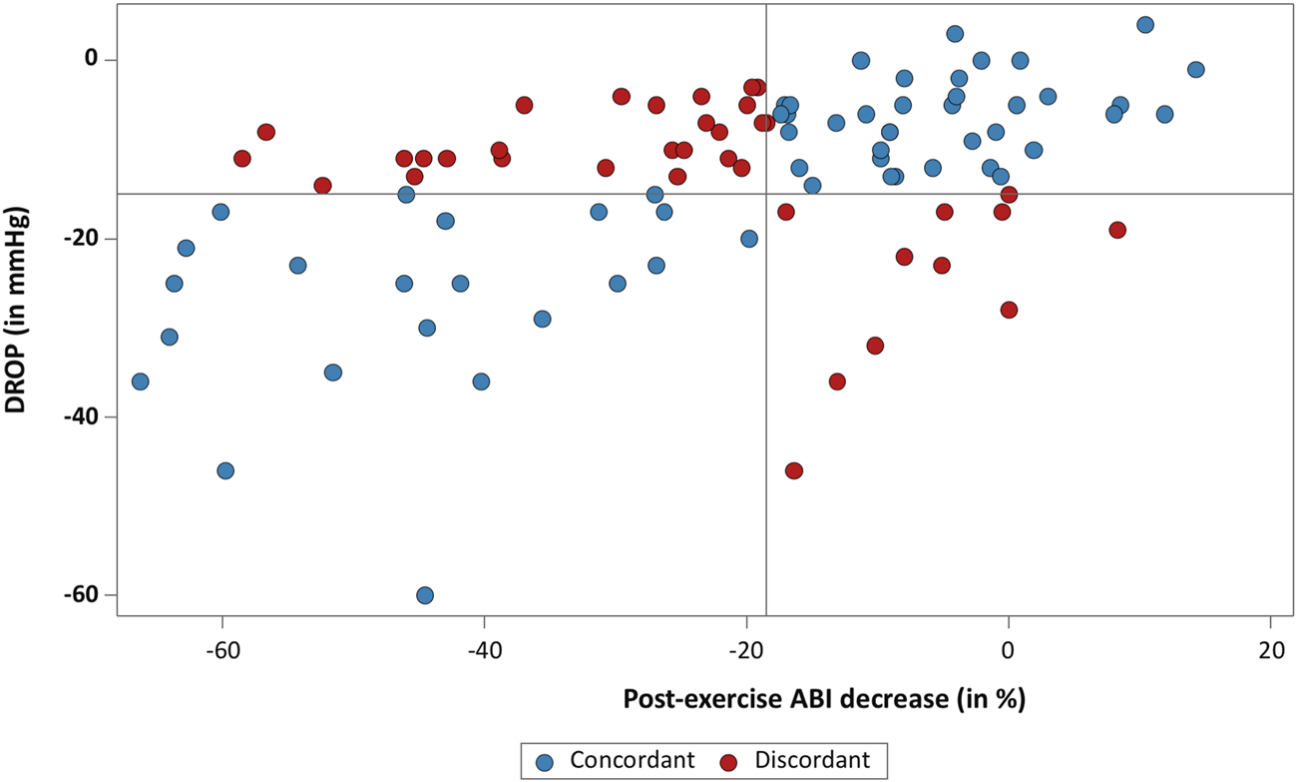
Results of Post-Exercise ABI and Exercise TcPO2 in patients with exertional limb symptoms and normal ABI (>0.90). Horizontal line represents the cut-off value (DROP: delta from rest of oxygen pressure) of exercise transcutaneous oxygen pressure measurement (−15 mmHg). Vertical line represents the cut-off value of Post-exercise ABI (ankle-brachial index) decrease (18.5%).

The prevalence of LEAD based on Post-Exercise ABI decrease was 50% whereas the prevalence was 34% based on Exercise-TcPO2 results. The agreement between both tests was weak with only a fair rating with a *k* value of 0.23[0.04–0.41] (Table 2). The characteristics of the concordant and discordant patients are presented in Table 3. Figure 2 depicts the results of both tests in the included population.

**Table 2:** Contingency table of both tests.

	Exercise TcPO2		Total
	Diseased*	Non diseased	
Post-Exercise ABI			
Diseased**	22	26	48
Non diseased	11	37	48
Total	33	63	96

Exercise TcPO2 means exercise transcutaneous oxygen pressure; Post-Exercise ABI means Post-exercise ankle-brachial index. *Means delta from rest oxygen pressure ≤−15 mmHg. ** means Post-exercise ABI decrease ≥18.5%.

**Table 3:** Characteristics of the concordant and discordant patients.

Clinical characteristics	Concordant n = 59	Discordant n = 37	P value
Age, years	58 ± 14	65 ± 10	<0.01
Male sex, no. (%)	43 (73%)	30 (81%)	0.36
Body mass index, kg/m^2^	26.0 ± 4.4	26.5 ± 4.8	0.62
Comorbidities, (history of), no. (%)			
Smoker (current or former)	45 (78%)	31 (84%)	0.39
Coronary disease	11 (19%)	14 (38%)	<0.05
Hypercholesterolemia	32 (54%)	26 (70%)	0.12
Diabetes	9 (15%)	10 (27%)	0.16
Peripheral artery disease*	10 (17%)	13 (35%)	0.04
Hypertension	29 (49%)	24 (65%)	0.13
Stroke	2 (4%)	4 (11%)	0.24
Current medications, no. (%)			
Statins	29 (49%)	20 (54%)	0.64
Anti-coagulants	4 (7%)	7 (19)	0.10
Antiplatelet	30 (51%)	28 (76%)	0.02
Others anticholesterolaemia	1 (2%)	0 (0%)	1.00
Angiotensin-converting enzyme inhibitors	12 (20%)	12 (32%)	0.18
Angiotensin II receptor antagonists	8 (14%)	6 (16%)	0.72
Ankle brachial index at rest (right)	1.05 [0.97–0.12]	1.05 [0.98–1.15]	0.66
Ankle brachial index at rest (left)	1.04 [0.98 ;1.16]	1.01 [0.90–1.15]	0.51
Maximal walking distance self-reported, m	500 [300–3000]	500 [200–2500]	0.72
Maximal treadmill walking distance (Exercise TcPO2), m	243 [121–525]	330 [149–524]	0.68
Maximal treadmill walking distance (Post-Exercise ABI), m	166 [107–524]	244 [137–524]	0.49

Results are presented mean ± standard deviation or median [25th ;75th], or number of observation (%). TcPO2 = Transcutaneous oxygen pressure measurement. Post-Exercise ABI = Post-exercise ankle-brachial index. Peripheral artery disease means history of revascularization (bypass/angioplasty/stent).

### Variables associated with the discordance between the exercise tests

Variables tested in the univariate analysis are presented in Table 4. Variables with a p value < 0.20 that were considered for the step-by-step logistic regression were gender, age, diabetes, dyslipidemia, hypertension, coronary disease, stent in the lower limbs, coronary artery bypass graft. Step-by-step logistic regression found that the presence of a stent in the lower extremities (OR: 5.85 [1.68;20.44; p < 0.01] and age (1.06 [1.01;1.11]; p < 0.05) were the variables independently associated with the discordance in this population (Table 4). Indeed, both tests had more risk to be discordant when a patient had an arterial stent or was old.

**Table 4:** Variables tested in the univariate and multivariate analysis.

Variables	Univariate analysis		Multivariate analysis	
	Odd ratio [IC95%]	p value	Odd ratio [IC95%]	p value
Gender		0.3619		
Women vs men	0.63 [0.23;1.71]			
Age	1.06 [1.02;1.11]	0.0059	1.06 [1.01;1.11]	0.0135
BMI		0.3470		
[18.5–24.99]kg/m^2^ vs <18.5 kg/m^2^	0.09 [0.00;4.14]			
≥25 kg/m^2^ vs <18.5 kg/m^2^	0.14 [0.00;6.13]			
Diabetes vs no diabetes	2.06 [0.75;5.68]	0.1635		
Dyslipidemia vs no dyslipidemia	1.99 [0.83;4.77]	0.1206		
Hypertension va no hypertension	1.91[0.82;4.45]	0.1340		
Tobacco status		0.3887		
Former vs never	1.88 [0.60;5.88]			
Active smoker vs never	1.08 [0.32;3.63]			
Family history of cardiovascular diseases vs no history	0.49 [0.12;1.94]	0.3103		
History of sleep apnea disorder vs no history of sleep apnea disorder	1.21 [0.26;5.76]	0.8077		
History of lumbar spinal stenosis vs no lumbar spinal stenosis	2.06 [0.63;6.70]	0.2292		
History of myocardial infarction vs no history	2.60 [1.02;6.61]	0.0443		
Lower limb arterial stent vs no history	6.60 [1.94;22.49]	0.0026	5.85 [1.68;20.44]	0.0056
Coronary arterial bypass graft vs no coronary arterial bypass graft	5.15 [1.27;20.89]	0.0218		
Carotid artery surgery vs no carotid artery surgery	3.31 [0.29;37.90]	0.3352		
Maximal walking distance on treadmill for Exercise TcPO2		0.2637		
[120–200]m vs ≤120 m	2.50 [0.63;9.90]			
[200–400]m vs ≤120 m	4.67 [1.01;21.65]			
>400 m vs ≤120 m	2.10 [0.58;7.57]			
Maximal walking distance on treadmill for Post exercise ABI		0.2453		
[120–200]m vs ≤120 m	1.82 [0.52;6.37]			
[200–400]m vs ≤120 m	4.53 [1.06;19.41]			
>400 m vs ≤120 m	1.89 [0.60;5.97]			

TcPO2 = Transcutaneous oxygen pressure measurement. Post-Exercise ABI = Post-exercise ankle-brachial index.

### Results of the healthy subjects

We included 37 healthy subjects that walked 15 minutes on the treadmill without any symptoms. The mean age of the healthy population was 64 +/−6 years old. There was a majority of men (62%) and mean body mass index was 23.9 +/−3.0 kg/m^2^. None of them was active smoker.

In this population the mean right and left ABI at rest was 1.19 +/−0.07 and 1.18 +/−0.08 respectively. The mean post-exercise ABI for the right leg and the left leg was 1.17 +/−0.11 and 1.16 +/−0.11, respectively. The mean post-exercise ABI decrease for the right and left legs were 1.8%+/−9.2% to and 1.1%+/−9.6%, respectively. The maximum post-exercise increase for the right leg was 15.2% and the maximal post-exercise decrease was 23.3%. The maximum post-exercise increase for the left leg was 15.7% and the maximal post-exercise decrease was 22.0%. Two right legs and two left legs had a value lower than >18.5% in this healthy population. Regarding Exercise TcPO2 values, the mean right DROP was −5 +/−3 mmHg whereas the mean left DROP was −6 +/−3 mmHg. The lowest DROP value was −13 mmHg in this healthy population and the maximal DROP value was 1 mmHg. No subject at the leg level had a DROP value lower or equal to −15 mmHg.

## Discussion

Post-exercise pressures are key elements for the diagnosis of LEAD in patients with exertional limb symptoms and normal ABI as suggested by the international recommendations^3,5^. One previous study has analyzed the concordance between exercise tests in a population of LEAD with or without abnormal ABI at rest^20^. However, no study has specifically studied the concordance between exercise tests in patients with normal ABI at rest. A good concordance between tests would suggest the tests are interchangeable, whereas discordance would indicate that a specific test is more suited to a specific patient population. To our knowledge, this is the first study to assess the concordance between Post-exercise ABI and Exercise-TcPO2 in patients with exertional limb symptoms and normal ABI. This study has demonstrated that the concordance between two exercise tests is only fair implying that Post-Exercise ABI cannot be replaced by Exercise-TcPO2 and *vice-versa*.

Post-exercise ABI is the most widely used test in clinical routine when ABI is normal at rest. However, there are no guidelines on how to perform the measurement^23,24^ and cut-off values have not yet reach consensus^7,8,23^. Mahe *et al*. have shown that approximately 1 in 5 patients were classified differently depending on which of the two AHA criteria was used^7^. Several authors have suggested that absolute Post-Exercise ABI decrease is less accurate than relative Post-Exercise ABI to diagnose LEAD^6,23^. One of the main advantages of Post-exercise ABI is that it is inexpensive and easy to perform in a clinical setting. Conversely, exercise-TcPO2 requires expensive equipment and dedicated software to perform the test^21,25^. Currently, only a few hospitals have this system available, although this may change in the near future as software to calculate the DROP freely available at no expense^25^.

The results of the current study suggest that there is discordance between the two tests used to diagnose LEAD. Indeed, we found a discordance between post-exercise ABI decrease >18.5% and the DROP value. This confirms previous results showing discrepancies between AHA post-exercise criteria and DROP value^20,26^. Several points can explain these discrepancies as suggested by the logistic regression. Age is one of the variables associated independently with the discordance in the present study. As Post-exercise ABI measures the variation of blood pressure in the main arteries, unlike Exercise-TcPO2 which measures the variation of transcutaneous oxygen pressure at capillary level^5,27^, arterial stiffness is a major source of error in ABI measurement. As shown in many studies, the main determinant of arterial stiffness is age^28,29^. Thus, discordance being prominent among older patients suggests that the very nature of the parameter being measured can explain the discrepancies. Furthermore, the presence of a stent was also associated with a discrepancy between the two tests. Proportionally, more patients considered as LEAD patients using Post-Exercise ABI (38.5%) than patients considered as suffering from LEAD using Exercise TcPO2 (18.2%) had a medical history of lower limb arterial stenting. One explanation could be that post-exercise ABI is difficult to assess in patients who are more severely affected by arterial disease, as it is when measuring ABI at rest^30^. Indeed, even with a marked on the skin it could be difficult to find the artery and detect arterial pressure^23,30^, additional delay in the measurement potentially resulting in inaccuracy. Also, the presence of a stent means the presence of stenosis. Stenosis can be a cause of a drop of downstream perfusion and pressure. A decrease of blood perfusion would affect both measurements while a drop of downstream pressure would only affect ABI measurement. A decrease of downstream pressure while perfusion is preserved occurs when a collateral arterial network is present. Thus, the discrepancies observed among patients with a medical history of arterial stenting could be due to the presence or not of a collateral arterial network. Apart from the variables found by the logistic regression several points can also explain the discrepancies. First, Exercise-TcPO2 is a measurement during exercise whereas Post-exercise ABI is a measure after the end of exercise. It has been suggested in animals and humans that exercise training induces collateral vessel development and enlargement during exercise^31–33^. This might explain the lower prevalence of LEAD found with Exercise-TcPO2 than with Post-exercise ABI in this study. Second, the Clark electrode only allows a small area of skin oxygen pressure measurement. We cannot exclude that this small area might have been well perfused by a collateral or inversely might have been poorly perfused by a partially occluded vessel. Third, systemic hypoxemia can modify Exercise-TcPO2 results whereas this will have no impact on Post-exercise ABI^34,35^. Additionally, several patients were closed to the cut-off values for Exercise-TcPO2 and Post-exercise ABI meaning that errors in diagnosis may be the consequence of each method precision.

The final interest of the present study is the results of the healthy subjects about Exercise-TcPO2 and Post-exercise ABI. Indeed, to the best of our knowledge, it is the first time that such results obtained with a treadmill test (3.2 km/h and 10% slope) that is currently used in clinical practice are presented. Using Post-exercise criterion, 4 legs out of 74 might be considered as positive for LEAD. However, it is important to underscore that the criterion of a post-exercise ABI decrease greater than 18.5% was validated in patients who experienced pain during walking that was not the case in these healthy subjects^8^. Furthermore, none of these healthy subjects who walked for 15 minutes had a DROP value lower than −15 mmHg suggesting that the DROP value previously validated is accurate^8,10,12,19^.

### Limitations

This study has several limitations. Firstly, the exercise tests were not randomized. This was due to time constraints during clinical practice. As a result, that rest conditions were not exactly the same in spite of the fact that the Post-exercise ABI walking test was performed after pain had disappeared and TcPO2 values had returned to baseline. Secondly, this study cannot confirm which test is the most accurate. Due to the fact that a disagreement of the magnitude detected was not expected, the computed tomography angiography data as a reference to determine tests accuracy was not acquired. Further work should be conducted to study this issue. Thirdly, it was not possible to assess the reliability of the tests in this study. We do not have our own reliability for the exercise tests but the intra-observer coefficient of variation (CV) for the ABI at rest in our vascular laboratory is 9.4% (typical error of the estimate is 0.06)^36^. Furthermore, the reliability of the exercise tests has been previously reported by several studies: i) Van Langen *et al*. showed that the inter-observer variability of the post-exercise ABI was 21%^37^; ii) intra-test and test-retest reliability of exercise TcPO2 using a similar protocol that we used has been reported by Abraham and colleagues as excellent^17,18^. Indeed, the correlation coefficient between two tests was r^2^ = 0.80 at distal level and intra-class correlation coefficients for the intra test-reliability and test-retest reliability were 0.920[0.846–0.967] and 0.807[0.723–0.868] respectively^17,18^.

## Conclusion

This study suggests that Post-Exercise ABI and Exercise-TcPO2 are not equivalent tests. The utility of each test in the management of LEAD must be defined in future studies.

## Methods

### Ethical standards

The data that support the findings of this study are available from the corresponding author upon reasonable request. The corresponding author had full access to all the data in the study and takes responsibility for its integrity and the data analysis.

### Study design and populations

Suspected lower extremity artery disease patients with normal ABI at rest and exertional limb symptoms:

This is a prospective, monocentric study on consecutive patients referred to the vascular unit of University Hospital, Rennes, France for exertional limb symptoms and normal ABI (>0.90).

The study was conducted from May 2016 to May 2019 and approved by an institutional review board (IRB). All participants gave written informed consent. The study protocol conforms to the ethical guidelines of the 1975 Declaration of Helsinki. The Exercise PAD study was registered with the American National Institutes of Health database under reference n° NCT03186391.

### Healthy subjects

Healthy subjects were analyzed to present the physiologic range of the responses of exercise tests. Subjects were contacted in our healthy database. To be included healthy subjects had to be able to complete a 15-minute walk on a treadmill (3.2 km/h, 10% slope) without any symptom. Exclusion criteria were:

-ABI < 1.00 or >1.40

-Presence of hypertension, heart failure, angina pectoris, diabetes, chronic obstructive pulmonary disease, supported by the presence of a medical treatment and the medical history;

-Presence of conditions likely to cause a functional limitation in walking and/or significant modification of physiological responses to the exercise: current or former smoker from less than 6 months, cancer (ongoing), Parkinson’s disease, renal failure (ongoing), supported by the presence of a medical treatment and the medical history.

-History of cardiovascular disease (heart failure, stroke, myocardial infarction) reported by the patient.

The part of this study including healthy subjects was conducted between January 2019 and December 2019 and approved by an institutional review board (CPP Ouest II- Angers, France). All participants gave written informed consent. The study protocol conforms to the ethical guidelines of the 1975 Declaration of Helsinki. The ELECTROPAD study was registered with the American National Institutes of Health database under reference n° NCT 03795103.

### Patient demographic characteristics

Variables collected included age, gender, body mass index, smoking status, comorbidities, and medications (statins, anti-hypertension treatment, antiplatelet, antidiabetic oral treatment or insulin).

### ABI measurement

After careful clinical evaluation, a measurement of ABI was performed according to AHA recommendations using a hand-held Doppler probe (8 MHz; Basic Atys Medical, Soucieu en Jarrest, France) by a trained vascular medicine physician^4^, with the exception of brachial blood pressures being measured using an automated oscillometric blood pressure monitor (Carescape Dinamap V100; GE Healthcare) in order to have the same procedure to measure the pressures at rest and after exercise and ensure almost simultaneous measures of upper and lower limb pressures^8^. The patient was at rest for 10 minutes in the supine position, relaxed, head and heels supported, in a temperature controlled room (21 °C)^30^. The following counterclockwise sequence was used: right brachial artery, right posterior tibial artery, right dorsalis pedis artery, left posterior tibial artery, left dorsalis pedis artery, left brachial artery, and right brachial artery. The ABI was calculated by dividing the highest pressure of the limb (dorsalis pedis or posterior tibial pressures) by the highest arm pressure as recommended^4,30^.

### Treadmill test

A treadmill test (3.2 km/h, 10% slope) was used for both the Exercise-TcPO2, which was performed first, and for the Post-Exercise ABI measurements as previously described by Stivalet and colleagues^8^. A minimum of 10 minutes was required between the two exercise tests. The patients were asked to inform the physician when and where (buttock, thigh, calf or other) the pain appeared^8^. Exercise was stopped for both studies according to patient limitation or up to a maximal distance of 525 m (over a period of 10 minutes)^8^.

### Exercise-TcPO2 test

Measurement of TcPO2 was performed using calibrated TcPO2 electrodes (TCOM/TcPO2; PF 6000TcPO2/CO2 Unit; Perimed; Jarfalla, Sweden). A reference electrode (chest electrode) was placed between the scapulae to measure systemic changes in TcPO2 during exercise^10–12,21^. One electrode was positioned on each calf. The measurement from the TcPO2 electrodes were used to calculate the Delta from Resting Oxygen Pressure (DROP) index, which was expressed in mmHg, and was the absolute change in TcPO2 from resting value in each of the 2 limb electrodes corrected for the absolute change in TcPO2 at the chest electrode. DROP was recorded in real time by the in-house Oxymonitor (version 2019.01.05) free Software (https://imagemed.univ-rennes1.fr/en/oxymonitor/download.php) as previously described^8,25^. As defined in previous studies, we considered a DROP ≤ −15 mmHg accurate to diagnose arterial stenoses of ≥50% assessed by computed tomography angiography (CTA) as a gold standard^8,10,12^.

### Post-exercise ABI

Two trained physicians performed these measurements: one at the brachial level with the automatic blood pressure device (Carescape Dinamap V100; GE Healthcare) and one at the limb level with the handheld Doppler. Post-Exercise brachial pressure was assessed on the same artery as it was for the ABI measurement at rest. When the resting ABI was measured, a black pen was used to mark the skin area where the highest limb pressure had been recorded with a hand-held Doppler^23,27^ in order to shorten the measurement process post exercise. As defined in our previous study, we considered Post-exercise ABI decrease ≥18.5% accurate to diagnose arterial stenoses of ≥50% assessed by computed tomography angiography (CTA) as a gold standard^8^.

### Statistical analyses

#### Data analysis

Continuous variables were described as mean ± standard deviation (sd) or median and interquartile range (IQR) values, and categorical variables were expressed as numbers (percentages). Baseline characteristics were compared between groups using *t*-test or U Mann-Whitney for quantitative variables, and chi-squared test or Fisher’s exact test were used for qualitative variables, depending on the distribution. Analysis was performed per patient, rather than per limb and only the symptomatic leg was included in the analysis. In case of both limbs symptomatic, the most symptomatic was analyzed. The concordance between Exercise-TcPO2 and Post-exercise ABI was assessed using the Kappa coefficient with a confidence interval of 95%. The Landis and Koch interpretation of kappa values was used: 0.21–0.40: fair; 0.41–0.60: moderate, 0.61–0.80: substantial; >0.80: almost perfect^38^. Logistic univariate regressions were performed first to define the variables independently associated with the discordance. Then variables with p value <0.20 were included in multivariate analysis. A backward stepwise procedure was used. A significance threshold of 0.05 was used in all statistical tests. Statistical analysis was performed using SAS 9.4 software (SAS Institute, Cary, NC, USA) (https://www.sas.com/en_us/home.html).
